# Obesity and hypertension in Asia: Current status and challenges

**DOI:** 10.1016/j.lanwpc.2021.100243

**Published:** 2021-08-05

**Authors:** Dinh-Toi Chu, Vijai Singh

**Affiliations:** 1 Center for Biomedicine and Community Health, International School, Vietnam National University, Hanoi, Vietnam; 2 Department of Biosciences, School of Science, Indrashil University, Rajpur, Mehsana, Gujarat, India

Obesity and hypertension are major risk factors for contracting non-communicable diseases (NCDs), ultimately leading to worldwide health issues and economic burden. Obesity tends to aggravate the chances of acquiring diseases such as type 2 diabetes, hypertension, fatty liver and cancer that in turn recedes both life expectancy and quality of life [1, 2]. Obesity and hypertension are the top risk factors for death globally (Fig. 1A) [3]. In the past decade, in Asia, obesity (Fig. 1B) and hypertension [4] have surged insurmountably. Li and colleagues published two repeated nationwide surveys in *The Lancet Regional Health - Western Pacific (10.1016/j.lanwpc.2021.100227)*, assessing the trends in prevalence of obesity and hypertension in China from 2007 to 2017 [5]. People aged 20 years and above from 31 provinces of mainland China were selected. Changes in blood pressure, body mass index (BMI), and waist circumference were assessed.

**Figure 1:**
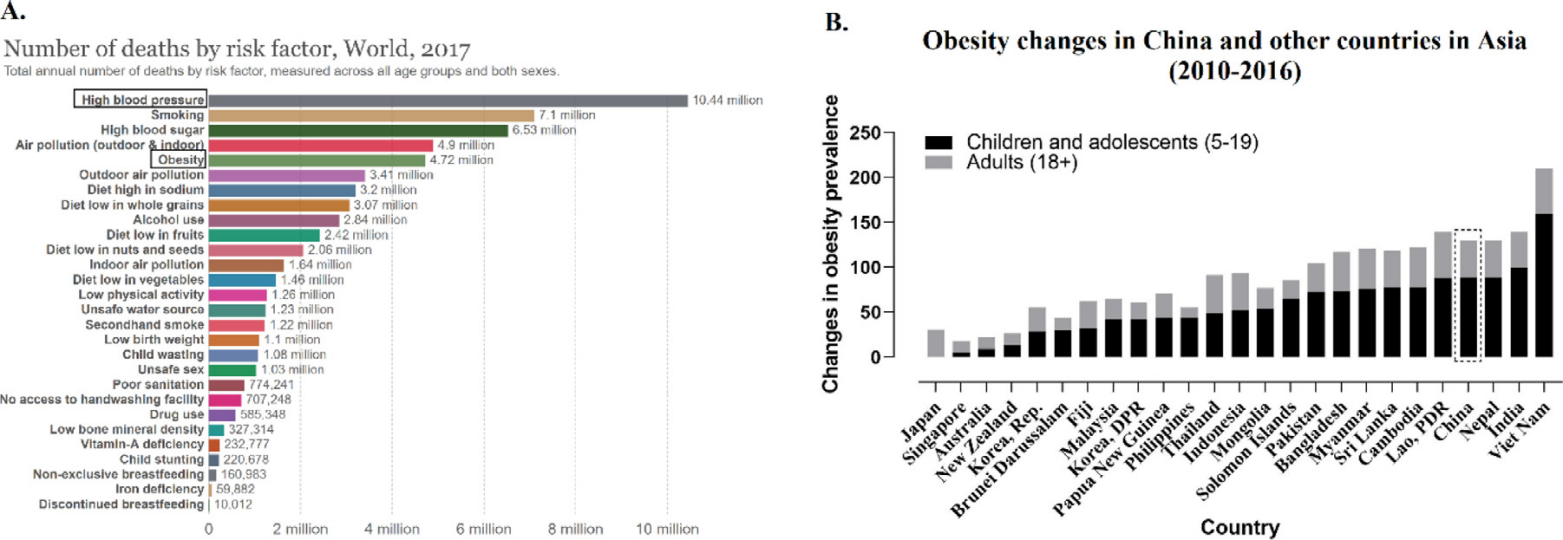
The effects of obesity and hypertension to the health. A. Hypertension and obesity are top risk factors for death in the world [3]; and B. The percent change in obesity prevalence in China and other countries in Asia from 2010 to 2016 (Source: WHO GHO 2020)

Prevention behavior for obesity and hypertension is strongly influenced by cognitive ability. However, cognitive-behavioral approaches for obesity and chronic diseases in China go largely unnoticed. Education in China is predominantly focused on academic results rather than health or physical development [6], which might be the reason behind the significant increase in the incidences of such diseases in China in recent years. In addition to cognitive problems, the tendency to switch from traditional foods to high-fat foods also has a major impact on these incidences [7]. Furthermore, technological development in tandem with decrease in physical activity has been another important contributing factor. Treatment of obesity with drugs or surgery is controversial and unpopular in China. This partially reflects the current clinical practice of the Chinese population. On the other hand, the findings have many similarities with several low-middle-income countries in Asia, but notably the trend of increasing incidence in developed countries seems to be markedly slower (Fig. 1B). According to WHO statistics, the trend of increase in the rate of obesity in China from 2007 to 2016 was 3.7-6.6%, which was roughly the same as Indonesia (3.9-6.9), but in Japan, the increase was significantly slower (3.0-4.4) [8]. Therefore, the experience from countries that have successfully eradicated obesity and other chronic diseases could be useful for China.

It was reported by Li and colleagues [5] that obesity and hypertension was significantly higher in adults in mainland China because of demographic confounding factors in 2017 as compared to 2007. A significant increase in mean BMI, waist circumference, systolic blood pressure (SBP) in adults was reported. In 2017, higher prevalence of high-normal blood pressure was observed amongst men, urban residents and young individuals. In China, rapid economic growth has brought along an unhealthy lifestyle and a high level of dietary sodium intake in their foods. Young adults exhibited increased SBP than aged adults that led to increased incidences of hypertension and future burden for cardiovascular diseases (CVD). Despite, there are limited reports and recommendations for hypertension management in the young generation in China [9]. In addition, the clinical significance of the treatment of hypertension in young individuals was questioned in the past, while hypertension was largely emphasized to be associated with aged individuals. China should establish a guideline and policy for preventing and controlling obesity by levying taxes on unhealthy foods and beverages. They should promote subsidized rates on healthy diets and foods. People should follow a healthy lifestyle and encourage regular exercise for fitness. More defined, focused and targeted intervention and preventive approaches are required to offset the increasing risk of CVD because of increase in obesity and hypertension.

The results of the study were mainly focused on describing rate change and the impact factors such as nutrition, physical activity were not mentioned, thus, the causal relationship has not been elucidated. In addition, the difference between the prevalence of obesity and hypertension in each province or economic region in China is an unanswered question. Furthermore, obesity and hypertension are often associated with other diseases, and the said study did not show the overview of those particular diseases. From the study, it is evident that the challenge of controlling obesity and hypertension is enormous in China, and so is in Asia. Changes in socio-economic status and lifestyle are leading to the co-existence of obesity and undernourishment in some Asian countries. Along with the increase in adult obesity, childhood obesity is also increasing rapidly. Asians are said to accumulate more visceral fat than Caucasians, and that is a major challenge because visceral fat is very difficult to reduce and it also adds to the risk of other diseases [10]. These challenges pose a need to improve the quality of management and treatment of obesity and hypertension in Asia, especially in China.

Summing-up, this study suggests that high-normal blood pressure is associated with higher risk of hypertension and CVD that may reduce life expectancy and lead to death. It was also found that higher prevalence of obesity and hypertension shifted from urban to rural populations over the time from 2007 to 2017. China has a large population living in rural areas and due to lack of consciousness, higher chances of obesity and hypertension in rural areas have led to increased incidence of NCDs. Therefore, with increase in SBP and waist circumference in rural populations, it is hinted that a large number of people are at high risk of acquiring hypertension in absence of effective preventive measurement and guideline.

## Declaration of Competing Interest

The authors declare no conflicts of interest.
